# Differentiating ICD-11 complex post-traumatic stress disorder from other common mental disorders based on levels of exposure to childhood adversities, the traumas of persecution and postmigration living difficulties among refugees from West Papua

**DOI:** 10.1192/bjo.2018.49

**Published:** 2018-08-24

**Authors:** Derrick Silove, Susan Rees, Mohammed Mohsin, Natalino Tam, Moses Kareth, Alvin Kuowei Tay

**Affiliations:** Director and Scientia Professor of Psychiatry, Psychiatry, Research and Teaching Unit, Liverpool Hospital, School of Psychiatry, University of New South Wales, Australia; Associate Professor, Psychiatry, Research and Teaching Unit, Liverpool Hospital, School of Psychiatry, University of New South Wales, Australia; Senior Statistician, Psychiatry, Research and Teaching Unit, Liverpool Hospital, School of Psychiatry, University of New South Wales, Australia; Project Manager, Psychiatry, Research and Teaching Unit, Liverpool Hospital, School of Psychiatry, University of New South Wales, Australia; Research Assistant, Psychiatry, Research and Teaching Unit, Liverpool Hospital, School of Psychiatry, University of New South Wales, Australia; National Health and Medical Research Council Early Career Fellow, Psychiatry, Research and Teaching Unit, Liverpool Hospital, School of Psychiatry, University of New South Wales, Australia

**Keywords:** Complex PTSD, common mental disorders, childhood adversities, postmigration living difficulties, refugees, trauma

## Abstract

**Background:**

Following years of controversy, a category of complex post-traumatic stress disorder (CPTSD) will be included in the forthcoming ICD-11.

**Aims:**

To test whether refugees with CPTSD differ from those with other common mental disorders (CMDs) in the degree of exposure to childhood adversities, adult interpersonal trauma and post-traumatic hardship.

**Method:**

Survey of 487 West Papuan refugees (response rate 85.5%) in Papua New Guinea.

**Results:**

Refugees with CPTSD had higher exposure to childhood adversities (CPTSD: mean 2.6, 95% CI 2.5–2.7 versus CMD: mean 1.15, 95% CI 1.10–1.20), interpersonal trauma (CPTSD: mean 9, 95% CI 8.6–9.4 versus CMD: mean 5.4, 95% CI 5.4–5.5) and postmigration living difficulties (CPTSD: mean 2.3, 95% CI 2–2.5 versus CMD mean 1.85, 95% CI 1.84–1.86), compared with those with CMDs who in turn exceeded those with no mental disorder on all these indices.

**Conclusions:**

The findings support the cross-cultural validity of CPTSD as a reaction to high levels of exposure to recurrent interpersonal trauma and associated adversities.

**Declaration of interest:**

None.

After a prolonged period of controversy surrounding the status of the category, complex post-traumatic stress disorder (CPTSD) will be included for the first time in the forthcoming revision of the ICD-11.^1^ According to ICD-11, full criteria for post-traumatic stress disorder (PTSD) must be met before considering an additional diagnosis of CPTSD. In addition, CPTSD can only be diagnosed following specified forms of trauma arising from interpersonal abuse and violence. The relevant trauma categories tend to be recurrent or chronic, for example, childhood sexual abuse, conditions of persecution and exposure to torture. In addition, the conditions (familial, social, political) surrounding and/or following these abuses tend to be unstable and stressful, potentially exacerbating the CPTSD reaction.^2^ ICD-11 defines CPTSD as a disturbance of self-organisation (DSO) manifesting as three symptom domains of affective dysregulation, negative self-concept and interpersonal difficulties,^1^ a response pattern that is likely to be associated with high levels of functional impairment.^3^

Preliminary studies in high-income countries offer broad support for the ICD-11 factor structure of CPTSD.^4^^–^^6^ In addition, two studies^7^ in low-income countries, including our first study among West Papuan refugees, indicate that CPTSD can be identified in culturally diverse, conflict-exposed communities.^8^ Further cross-cultural research is needed to establish the universal applicability of CPTSD. We examine this issues in refugees from West Papua who have not been exposed to international constructs of traumatic stress or conventional mental health services. Specifically, we investigate whether CPTSD is associated with high levels of exposure to childhood abuse and adversity, the traumas of persecution and postmigration living difficulties (PMLDs). The comparison groupings are compatriots with one or more other common mental disorders (CMDs) and those with no mental disorder. We also examine whether CPTSD is associated with high levels of psychosocial dysfunction in comparison with those with CMDs.

## Method

### Setting

West Papuans have experienced a prolonged period of persecution since the Indonesian annexation of their homeland in 1969.^9^ To quell the low-grade armed-resistance movement, the Indonesian military has committed widespread atrocities including torture, disappearances, extrajudicial imprisonment and mass displacement of populations.^10^ Successive waves of refugees have crossed into neighbouring Papua New Guinea (PNG), the largest concentration resettling in Kiunga, a town near the border. Refugees located in Kiunga confront a range of stressors and deprivations, including risk of incursions by hostile elements from across the border; geographical isolation; harsh living conditions including food and water shortages related to intermittent floods and droughts; few employment opportunities; and lack of services including in health. The population has had no formal mental health assistance and as such, are not familiar with international diagnostic traditions or Western constructs of trauma.

### Participants

Our team has worked with West Papuan refugees in Australia and in PNG for over a decade, a period in which we have established strong ties and a foundation of trust with the community. West Papuan refugees play an integral role in all our studies and interventions. Prior to our study, our team made several visits to Kiunga to consult widely among the West Papuan community regarding the focus and potential value of the proposed survey. The survey was initiated only after a consensus was reached in the community that the information generated would be of benefit in identifying the mental health needs of the population.

The study was conducted between March and September 2016. A PNG government census of all West Papuans residing in the country had been completed the year prior to our study. Nine settlements were identified as the sites of residence of almost all West Papuans in Kiunga. We confirmed the composition of all households in these nine villages by undertaking a house-to-house survey prior to our main study (see supplementary File 1 available at https://doi.org/10.1192/bjo.2028.49). For the purposes of our study, we defined West Papuans as native born to the territory or the offspring of at least one West Papuan parent.

Although our survey included adolescents, the present analysis is restricted to adults aged 18 years and older. All West Papuans with whom we made direct contact agreed to participate in the study. Those present identified a further 157 (18.3%) adults as regular residents but who were travelling in other parts of PNG during the entire course of our survey. Our final analytic sample comprised 487 adults (response rate 100% of those present, or 81.7% if absentees were included).

### Measures

#### Diagnoses

The Refugee Mental Health Assessment Package (R-MHAP)^11^ was developed for use among the West Papuan population during a previous study in Port Moresby, the capital of PNG (see supplementary File 2). Responses are recorded on an electronic tablet. The package includes modules recording trauma exposure, PMLDs and a range of psychosocial issues. In addition, the R-MHAP assess several CMDs based on DSM-IV/DSM-5 and ICD-10/ICD-11 formulations^12^^–^^15^ Symptoms are rated on a four-point severity scale; the highest two severity ratings are required for a categorical endorsement of the symptom. In all interviews, participants are required to complete the full symptom list for each disorder without skip rules. This allows analysis according to both diagnostic categories, for example, using DSM algorithms; or as dimensional scores in which a symptom count is derived by adding the severity ratings for each symptom.

Previous psychometric analysis of the R-MHAP indicated sound levels of interrater and test–retest reliability for diagnostic assignments.^11^ In addition, there was sound agreement between the R-MHAP and an independent diagnostic assessment using the Structured Clinical Interview (SCID)^16^ in distinguishing respondents with and without a mental disorder, thereby supporting the convergent validity of the instrument. Individual diagnoses also showed a consistent pattern of stability over a 6-month period. For the present study, we selected CMDs most commonly identified among refugees, that is, major depressive disorder, generalised anxiety disorder, panic disorder, persistent complicated bereavement disorder, separation anxiety disorder and intermittent explosive disorder.

We used the DSM-5 format to ensure consistency in defining and analysing diagnostic categories. Given that the DSM system does not include a category of CPTSD, we followed a series of steps to render the ICD-11 descriptive account of the disorder in the format of the DSM-5. We operationalised the trauma events identified by ICD-11 to specify the entry criterion for CPTSD. In relation to symptoms of CPTSD, the first step involved the research team formulating and refining relevant ICD-11 items via an iterative process of consultation, modification and feedback. The aim was to produce simple items that captured the essential features of each of the dimensions of the disorder. The draft symptoms were then subjected to a systematic qualitative cross-cultural assessment (consistent with the procedure used for all other diagnoses) based on consultations with psychiatrists who share the Melanesian culture with West Papuans, followed by individual interviews and focus groups with members of the West Papuan community. Our final CPTSD module includes six CPTSD (or DSO) symptoms (two each for affective dysregulation, negative self-concept and interpersonal problems) (see supplementary File 5 for CPTSD items). As indicated, to make the diagnosis, criteria for PTSD also had to be met.

#### Childhood adversities

Early childhood adversities were assessed using a locally adapted version of the Adverse Childhood Experiences International Questionnaire (ACE-IQ).^17^ The ACE-IQ was developed by the World Health Organization (WHO) to assess a wide range of childhood adversities occurring prior to 18 years of age, including family dysfunction; physical, sexual and emotional abuse; neglect by parents or caregivers; peer violence; witnessing community violence; and exposure to collective violence. The ACE-IQ has been used in large-scale epidemiological studies worldwide.^18^ A Pearson's correlation matrix was generated to assess the extent to which individual childhood adversity items were interrelated (Table 1). Physical, emotional and sexual abuse were highly correlated (all *r*  ≥0.93), prompting us to collapse these items into one index. For the analysis, therefore, we used three childhood adversity indices (CA.1 a composite of physical/emotional/sexual abuse; CA.2 community violence; CA.3 peer violence).

**Table 1:** Pearson correlation matrix of interrelated forms of childhood adversities (CAs) (*n* = 487)

	Physical and emotional abuse	Sexual abuse	Community violence	Peer violence
CA.1 Physical and emotional abuse^a^	1.00			
Sexual abuse^a^	0.41*	1.00		
CA.2 Community violence	0.08	0.10*	1.00	
CA.3 Peer violence	0.50*	0.21*	0.17*	1.00

a. Physical/emotional and sexual abuse items were combined to generate a composite index of emotional/sexual abuse (CA.1).

* *P* < 0.05.

#### Traumatic events associated with persecution and war

We identified 31 types of traumatic events that were anchored to key historical epochs of persecution, conflict and displacement since the Indonesian annexation of West Papua (spanning the period 1960 through to 2010). The items were compiled following an established procedure of extensive consultation with the West Papuan refugee community in Kiunga to identify, refine and contextualise these experiences. The traumatic events included in our final inventory related primarily to gross human rights violations such as deliberate destruction of property, mass displacement, torture, physical assaults, extrajudicial murders, combat-related violence, family deaths, witnessing violence, extreme lack of medical care in times of emergency and intimate partner violence. We generated a mean traumatic event score based on whether refugees had ever experienced items on the list (scored 1 if yes, 0 if no). Our approach was guided by past experience in conducting cross-cultural studies that indicated that errors in recall increased if efforts were made to date or quantify exposure to each type of trauma in societies that were non-numeric, gave low priority to recording dates, and had high levels of exposure to multiple traumas.

#### PMLDs

An inventory based on the Humanitarian Emergency Settings Perceived Needs scale^19^ was used to assess the prevalence of common forms of stressors in the community. We adapted the measure to the local culture and context by following an extensive series of consultations in which all items were evaluated in focus groups and by individual informant interviews undertaken with members of the Kiunga community. The measure comprises 26 items rated on a four-point scale (scored 0,  not a problem; 1, a bit of a problem; 2, a moderately serious problem; 3,  a very serious problem). Examples of PMLDs include shortages of food, water, shelter, toilets and clothing; limited opportunities to pursue livelihoods; problems with safety; and poor access to information and aid. We generated a mean PMLD index based on the addition of individual item scores (range for each item, 0–3).

#### Functional impairment

We assessed functional impairment using the abbreviated version of the WHO Disability Assessment Schedule (WHODAS 2.0), based on the International Classification of Functioning and Health.^20^ The WHODAS assesses six core domains including cognition/communication; mobility; self-care; interpersonal interaction; life activities; and participation in society. Items are rated on a five-point Likert scale: none, 1; mild, 2; moderate, 3; severe, 4; extreme, 5 (see supplementary File 2). We generated a total mean score based on the sum of scores of WHODAS items.

#### Field team and procedure

Interviews were conducted by a field team drawn from the West Papuan community and managed by a West Papuan refugee (M.K.) who has worked with our research team on similar projects for several years (see supplementary File 3).

### Ethics

Ethical permission for the study was provided by the University of New South Wales Human Research Ethics Committee and the Medical Research Council of PNG Ethics Committee. Signed consent was obtained from all participants under conditions of privacy after clarifying that the decision to participate in the research was entirely voluntary.

### Statistical analysis

Descriptive statistics were computed to assess the prevalence of all indices. We applied diagnostic algorithms to derive cases meeting criteria for all disorders measured. To generate sufficient cell sizes, we compared participants with CPTSD with those with one or more CMDs; the remainder forming the no mental disorder group. We applied MANOVA to compare the three diagnostic groupings on the three derived indices of childhood adversity, mean trauma count and the mean PMLD count.

We also derived both a mean CPTSD symptom score (that is, including all symptoms from the three PTSD and three DSO domains) and then a mean DSO symptom score alone (excluding PTSD). The mean score for each was based on adding the severity score of all symptoms (1,  not at all; 2,  a little bit; 3,  quite a lot; 4, extremely) and dividing by the number of items. The CPTSD and DSO symptom scores were then used as dependent variables in two stepwise, multiple linear regression analyses in which predictor variables were childhood adversity, trauma count and PMLDs (see supplementary File 3).

## Results

### Sociodemographic characteristics

The sociodemographic characteristics of the sample are reported in Table 2. The sample included 487 adults (men 55.9%) and the mean age of the whole sample was 35.8 (s.d. = 0.65) years. Half the sample (46.6%) had completed primary school education, and one in ten (9.5%) held post-school degrees or certificates. Over half (56.9%) originated from West Papua, the remainder (43.1%) were born to West Papuan parents in PNG. The West Papuan born individuals had lived in Kiunga for a mean of 15.6 (s.d. = 0.48) years. The majority (62.6%) had indeterminate status as displaced people, 14.6% were PNG citizens and 22.8% held permissive residency status that conferred the right to remain in PNG with some restrictions. Supplementary File 4 detail rates of exposure to childhood adversities, traumatic events and PMLDs.

**Table 2:** Sociodemographic characteristics of the West Papuan Kiunga community sample (*n* = 487)

Sociodemographic variables	
Age, years: mean (s.d.)	35.81 (0.65)
Gender, *n* (%)	
Men	272 (55.9)
Women	215 (44.1)
Educational attainment, *n* (%)	
None	28 (5.8)
Completed primary	227 (46.6)
Completed secondary	186 (38.2)
Completed university/college	46 (9.5)
Residency status, *n* (%)	
Refugee	305 (62.6)
Permissive Residency Status	111 (22.8)
Citizen of Papua New Guinea	71 (14.6)
Country of birth, *n* (%)	
West Papua	277 (56.9)
Papua New Guinea	210 (43.1)
Time of residency in Kiunga, years: mean (s.d.)	15.57 (0.48)

### Prevalence of CPTSD and CMDs

The prevalence for individual mental disorders were as follows: PTSD, 59 (12.1%) (not taking into account CPTSD); CPTSD, 45 (9.3%), noting that only 2 individuals (0.4%) met criteria for PTSD without additional CPTSD, that is, virtually all respondents with PTSD also experienced CPTSD. This pattern of findings precluded any comparison between the PTSD and CPTSD groupings.

In relation to other CMDs, 273 (56.2%) had major depressive disorder; 129 (26.5%) generalised anxiety disorder; 39 (8.0%) panic disorder; 48 (9.9%) persistent complicated bereavement disorder; 48 (9.9%) separation anxiety disorder; and 27 (5.6%) intermittent explosive disorder. Almost two-thirds (64.4%, *n* = 29) of survivors with CPTSD were born in West Papua, which is consistent with the history of high exposure to persecution and conflict in the home country. In relation to comorbidity, almost all those with CPTSD had two or more other current CMDs (*n* = 44, 97.8%) compared with fewer than half of individuals in the CMD group (*n* = 111, 44%) (χ^2^(1) = 144.18, *P* < 0.01).

### Comparisons of childhood adversities, traumatic events, PMLDs and functional impairment across all groups

MANOVA indicated an overall difference in the pattern of childhood adversities, traumatic events, PMLDs and functional impairment across the three diagnostic groupings (Wilks Λ = 0.93, *F*(5,30) = 1.05, *P* ≤ 0.001).

**Fig. 1:**
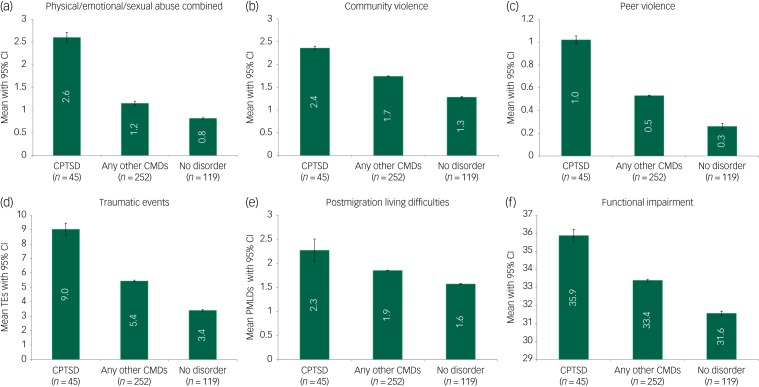
Comparisons between complex post-traumatic stress disorder (CPTSD), common mental disorders (CMDs) and no disorder groups in prevalence of childhood adversities ((a) combined physical/emotional/sexual abuse; (b) community violence; (c) peer violence), (d) traumatic events (TEs), (e) postmigration living difficulties (PMLDs) and (f) functional impairment.

Table 3 and Fig. 1 provide a detailed analysis of the mean differences in childhood adversities, traumatic events, PMLD and functional impairment in two-way contrasts involving the CPTSD, CMD and no disorder groupings. In relation to the key comparison between the CPTSD and CMD groupings, after adjusting for sociodemographic variables, the CPTSD group reported significantly higher mean scores on childhood adversities (CPTSD: mean 2.6, 95% CI 2.5–2.7 versus CMD: mean 1.15, 95% CI  2.5–2.7); traumatic events (CPTSD: mean 9, 95% CI  8.6–9.4 versus CMD: mean 5.4, 95% CI 5.4–5.5); PMLDs (CPTSD: mean 2.3, 95% CI 2–2.5 versus CMD: mean 1.85, 95% CI 1.84–1.86); and functional impairment (CPTSD: mean 35.9, 95% CI 35.5–36.2 versus CMD: mean 33.4, 95% CI 33.3–33.5) (Wilks Λ = 0.85, F(6, 285) = 8.58, *P* ≤ 0.001).

**Table 3:** Association of sociodemographic characteristics, childhood adversities, traumatic events, postmigration living difficulties and functional impairment with complex post-traumatic stress disorder (CPTSD), any common mental disorders (CMDs) and no disorder in West Papuan refugees from Kiunga, Papua New Guinea^a^

Characteristic and comparison groups	
Age, mean (95% CI)	
CPTSD (*n* = 45)	39.7 (39.0–40.4)
Any other CMDs (*n* = 252)^b^	37.0 (36.9–37.1)
No disorder (*n* = 119)	32.6 (32.4–32.8)
*Childhood adversities*, *mean (95% CI)*	
Physical/emotional/sexual abuse combined (mean)	
CPTSD (*n* = 45)	2.6 (2.5–2.7)
Any other CMDs (*n* = 252)	1.15 (1.10–1.20)
No disorder (*n* = 119)	0.82 (0.79–0.85)
Community violence (mean)	
CPTSD (*n* = 45)	2.36 (2.32–2.40)
Any other CMDs (*n* = 252)	1.74 (1.73–1.75)
No disorder (*n* = 119)	1.28 (1.26–1.30)
Peer violence (mean)^c^	
CPTSD (*n* = 45)	1.02 (0.99–1.05)
Any other CMDs (*n* = 252)	0.53 (0.52–0.54)
No disorder (*n* = 119)	0.26 (0.23–0.29)
Traumatic event exposure, mean (95% CI)	
CPTSD (*n* = 45)	9.0 (8.6–9.4)
Any other CMDs (*n* = 252)	5.4 (5.4–5.5)
No disorder (*n* = 119)	3.4 (3.3–3.5)
Postmigration living difficulties, mean (95% CI)	
CPTSD (*n* = 45)	2.3 (2.0–2.5)
Any other CMDs (*n* = 252)	1.85 (1.84–1.86)
No disorder (*n* = 119)	1.57 (1.56–1.58)
Functional impairment, mean (95% CI)	
CPTSD (*n* = 45)	35.9 (35.5–36.2)
Any other CMDs (*n* = 252)	33.4 (33.3–33.5)
No disorder (*n* = 119)	31.6 (31.4–31.7)
*Gender, n (%) 95% CI*	
CPTSD (*n* = 45)	
Men	26 (57.8) 43.3–72.2
Women	19 (42.2) 27.8–56.7
Any other CMDs (*n* = 252)	
Men	140 (55.6) 49.4–61.7
Women	112 (44.4) 38.3–50.6
No disorder (*n* = 119)	
Men	63 (52.9) 44.0–61.9
Women	56 (47.1) 38.1–56.0
*Country of birth, n (%) 95% CI*	
CPTSD (*n* = 45)	
West Papua	29 (64.4) 50.5–78.4
Papua New Guinea	16 (35.6) 21.6–49.5
Any other CMDs (*n* = 252)	
West Papua	153 (60.7) 54.7–66.7
Papua New Guinea	99 (39.3) 33.3–45.3
No disorder (*n* = 119)	
West Papua	57 (47.9) 38.9–56.9
Papua New Guinea	62 (52.1) 43.1–61.1

a. We excluded the cases of 70 individuals because of missing data on symptom variables.

b. We derived a composite category of any other CMDs comprising individuals (*n* = 252) who met any other threshold criteria for at least one of the remainder disorders (other than CPTSD): post-traumatic stress disorder, PTSD (without CPTSD disturbance of self-organisation symptoms), depression (MDD), generalised anxiety disorder (GAD), panic disorder, persistent complicated bereavement disorder (PCBD), separation anxiety disorder (SAD) and intermittent explosive disorder (IED).

c. CPTSD case assignments were defined as having at least one symptom of each of the CPTSD domains including intrusion, avoidance, hyperarousal, affective dysregulation, negative self-concept and international dysfunction. Showed significant (P < 0.05) stepwise linear association with CMD groups.

Both the CPTSD group and the CMD groups scored higher on all four indices compared with the no disorder group (CPTSD versus no disorder grouping: Wilks Λ = 0.61, *F*(6, 152) = 16.38, *P* ≤ 0.001; CMD versus no disorder grouping: Wilks Λ = 0.89, *F*(6, 404) = 8.11, *P* ≤ 0.001).

In summary, the results indicated a regular, stepwise association in relation to exposure to childhood adversities, traumatic events, PMLDs and functional impairment across the three groupings; the CPTSD grouping consistently reported the highest levels on all these variables, followed by the CMD grouping, and at the lowest endorsement, the no disorder grouping.

### Stepwise, linear hierarchical regression analysis

A mean CPTSD symptom score was computed based on the addition of all item severity scores (rated 0–3). Based on the whole sample, stepwise hierarchical regression analysis was conducted in which blocks of explanatory variables were entered in sequential steps as follows: (a) sociodemographic variables (age, gender, marital status, employment); (b) childhood adversities based on indices found to be associated with CPTSD in the preceding bivariate analyses as follows: CA.1, physical/emotional/sexual abuse; CA.2, community violence; CA.3, peer violence; (c) mean traumatic event exposure (the average of positively endorsed trauma items); and (d) the mean PMLD score (the average of positively endorsed PMLD items).

At step 1, none of the sociodemographic variables predicted the CPTSD score (*F*(4, 481) = 1.44, *P* = 0.22) (*R*^2^ = 0.003). Of all the substantive variables, only the family dysfunction item in the childhood adversity measure failed to contribute (*P* > 0.20) (in step 1).

At step 2, CA.1 (physical, emotional and sexual abuse), CA.2 (community violence) and CA.3 (peer violence) all predicted the CPTSD score (*F*(8, 477) = 10.66, *P* < 0.001, β = 0.06, *P* < 0.001) (*R*^2^ = 0.13). At step 3 (*F*(8, 477) = 10.66, *P* < 0.001) the predictors of the CPTSD score included CA.1 (physical, emotional and sexual abuse) (β = 0.06, *P* < 0.001), CA.3 (peer violence) (β = 0.12, *P* < 0.001) and traumatic events (β = 0.24, *P* < 0.01) (*R*^2^ = 0.18). At step 4 (*F*(9, 476) = 12.58, *P* < 0.001), CA.1 (physical, emotional and sexual abuse) (β = 0.05, *P* < 0.001), CA.3 (peer violence) (β = 0.11, *P* < 0.001), traumatic events (β = 0.19, *P* = 0.33) and PMLDs (β = 0.16, *P* < 0.01) were each associated with CPTSD (*R*^2^ = 0.19). CA.2 (community violence) no longer was a predictor of the CPTSD score after including traumatic events and PMLDs.

In summary, the hierarchical linear regression analysis showed that at steps 3 and 4, a combination of childhood trauma and adversity, traumatic events associated with persecution and displacement and PMLDs together predicted CPTSD symptoms.

We then repeated the multiple regression analyses applying as the dependent variable the mean score of the three DSO symptoms only (that is, excluding PTSD symptoms). Exposure to childhood adversities of emotional and sexual abuse (β = 0.10, *P* < 0.001) and peer violence (β = 0.10, *P* < 0.01), traumatic events (β = 0.24, *P* = 0.012) and PMLDs (β = 0.16, *P* < 0.001) were all significant predictors of DSO symptoms. The fit statistics for the hierarchical linear regression models are shown in Table 4.

**Table 4:** Hierarchal linear regression models assessing the contributions of sociodemographic variables, childhood adversities, traumatic events and postmigration living difficulties based on the entire sample (*n* = 487)

HLR models of predictors of complex post-traumatic stress disorder	Degree of freedom	Sum of squares	Mean squares	*R*^2^	Adjusted *R*^2^
Step 1: age, gender, employment, marital status	4	1.34	0.33	0.01	0.003
Step 2: age, gender, employment, marital status; CA.1, CA.2, CA.3	7	15.81	2.26	0.14	0.13
Step 3: age, gender, employment, marital status; CA.1, CA.2, CA.3; traumatic events	8	21.76	91.50	0.19	0.18
Step 4: age, gender, employment, marital status; CA.1, CA.2, CA.3; traumatic events; postmigration living difficulties	9	21.76	2.42	0.19	0.18

CA, childhood adversities; CA.1, emotion/sexual abuse (emotional and sexual abuse were combined to form a composite variable in the hierarchal linear regression analyses); CA.2, community violence; CA.3, peer violence.

## Discussion

For the newly adopted ICD-11 diagnosis of CPTSD to make an added contribution to the refugee mental health field, it is important to demonstrate its unique properties. A key question is whether, in keeping with theory, people with CPTSD are distinguished from those with other CMDs in the extent of their exposure to childhood abuse and adversity, the traumas of persecution and conditions of post-traumatic adversity. In addition, it would be expected that CPTSD is associated with high levels of functional impairment. Our study indicates that refugees with CPTSD report higher levels of exposure to the triad of childhood adversities, traumatic events and PMLDs compared with those in the CMD grouping. The pattern of findings indicates a hierarchy of adversity in which individuals with CPTSD experience the highest levels, followed by people with CMDs alone and finally, those with no disorder. The stepwise pattern of association remained consistent for comorbidity and functional impairment, in that those with CPTSD reported the most extreme pattern on both these indices. The multiple linear regression analysis further supported a cumulative adversity model of CPTSD in which each domain (childhood adversity, adult interpersonal trauma, PMLDs) contributed to the symptom score. The findings therefore add to the cross-cultural validity of the newly formulated ICD-11 construct of CPTSD.

### Strengths and limitations

We applied a rigorous sampling approach, drawing both on recent census data and a comprehensive preliminary survey to identify all in-scope individuals in the catchment area. We achieved a high response rate (85.8%) even accounting for residents absent from the catchment area during the course of the study. Although Kiunga is the largest settlement of West Papuans in PNG, restriction of the sample to one location limits the generalisability of our findings. We undertook a systematic qualitative and quantitative approach to developing our assessment procedure but the risk of transcultural error in measurement can never be entirely discounted. Only a small number of participants had PTSD without CPTSD, preventing us from comparing the two categories directly.

We note that ICD-11 requires that PTSD is present to make an additional diagnosis of CPTSD, an *a priori* hierarchical structure that presents challenges in comparing the two categories as independent entities. It is possible, therefore, that this hierarchical structure will obscure the presence of PTSD related to other causes, for example, civilian accidents. We note that other formulations do not mandate the presence of PTSD to make a diagnosis of complex traumatic stress.^21^ Although a diagnosis of both PTSD and CPTSD requires exposure to at least one relevant traumatic event, this definitional requirement does not explain the large difference in trauma exposure rates observed between the CPTSD and CMD groupings. Retrospective reporting, for example of childhood adversities and traumatic events, may be subject to bias in both directions; for example, PTSD symptoms of amnesia may lead to underreporting of past events, whereas flashbacks and other intrusions may accentuate trauma memories.

### Implications for the construct validity of CPTSD

Caveats notwithstanding, we were able to identify CPTSD in almost one in ten (9.3%) refugees participating in our survey. The rarity of PTSD on its own raises a question of whether the expanded formulation of CPTSD is more relevant to assessing the impact of sequential traumas of persecution among refugees in general.

As indicated, past studies into CPTSD have largely focused on civilian or military populations.^8^^,^^22^ Our findings add to early but growing evidence that CPTSD can be identified across cultures, in this case, in a population that has had no prior sensitisation to Western notions of traumatic stress or formal psychiatric diagnostic traditions. The history of West Papuan refugees supports the ecological validity of our findings. Two-thirds of individuals who met criteria for CPTSD were born in West Papua where exposure to persecution and conflict was pervasive, whereas those born in PNG were protected to some extent from these experiences (noting, however, that the distinction is not absolute in that it is common for people born in PNG to return to the homeland, placing them at some risk). Nevertheless, this difference in the location in which participants grew up (one group in an environment of prolonged exposure to persecution and human rights violations, the other in a place of relative safety) adds support to the notion of CPTSD as a response to chronic or recurrent conditions of interpersonal threat and violence.

### The relevance of CPTSD in refugee mental health

Our findings suggest that the refugee mental health field may need to shift ground in its diagnostic tradition from a singular focus on PTSD to one that incorporates the additional features of CPTSD, a step that has implications for psychological interventions applied to that population. For example, there may be benefit in broadening the scope of brief psychotherapies to include more explicit elements of DSO, that is, to support strategies to achieve affect regulation, overcome negative self-appraisals such as guilt and shame, and modulate interpersonal interactions, strategies that need to be grounded in culturally sensitive approaches.^23^^,^^24^ Rehabilitation programmes may be needed for those who have experienced the most extensive traumas related to human rights violations, given the high rates of functional impairment recorded among refugees with CPTSD. More generally, an ecological approach that reduces the impact of postmigration stresses may assist in preventing the elaboration of the traumatic stress response following exposure to repeated trauma and conditions of adversity.^25^

Creating conditions of safety and security, meeting basic survival needs (clean water and reliable food supplies), providing essential services (in health and education), and creating opportunities for work and education, provisions that are all lacking in the Kiunga area, may play an important role in mitigating symptoms of CPTSD and other CMDs. In relation to epidemiological studies, public health screening and clinical practice, there may be reason to add the DSO criteria to identify a subpopulation in the refugee and post-conflict mental health field with high levels of functional impairment.

In summary, our study offers the first evidence that compared with refugees with other CMDs and those with no mental disorders, individuals with CPTSD report a high level of exposure to the triad of childhood adversities, the traumas of persecution and PMLDs. Nevertheless, to build support for the universal applicability of CPTSD, our findings need to be confirmed among other refugee groups from a diversity of cultures.

## Supplementary material

For supplementary material accompanying this paper visit http://dx.doi.org/10.1192/bjo.2018.49. click here to view supplementary material
