# Supplementary material for: Differentiating ICD-11 complex post-traumatic stress disorder from other common mental disorders based on levels of exposure to childhood adversities, the traumas of persecution and postmigration living difficulties among refugees from West Papua
Source: BJPsych Open. 2018 Aug 24;4(5):361–7. doi: 10.1192/bjo.2018.49 (PMC6436047; doi:10.1192/bjo.2018.49)
Supplement: Supplementary file 1 [file S2056472418000492sup001.docx]

**Supplementary file 1: sampling**

We based our sampling frame on census data collected a year earlier by the PNG government documenting the whereabouts of all West Papuans living in the country. We confirmed the census findings by reference to local informants (The United Nations High Commissioner on Refugees (UNHCR), community leaders, officials in the local government, and workers in faith-based services) who reported that virtually all West Papuans in the catchment area resided in the nine settlements of Nemo Corner, Defence Camp, Kungim, Michael Corner, Bun Settlement, Kumbit 1 and 2, Komoka Corner, Selamat Corner, and Waterfront. To update the census data, we conducted a preliminary full door-to-door household survey of the nine designated villages, mapping the current composition of each dwelling. Eligible participants included all adults (18 years and older) and adolescents (11-17 years) born in West Papua or, if born in PNG, having at least one parent born in West Papua.

**Supplementary file 2: adaptation and translation of survey assessment tools**

Details of the qualitative and psychometric steps taken to develop, culturally adapt and test the R-MHAP have been provided previously and will only be summarized here (1). We followed a sequential process of consultation with community members and PNG psychiatrists to test the cultural and contextual relevance of the mental health constructs under study, a process made possible by the commonalities in ethnicity and culture of populations from PNG and West Papua. Focus groups included West Papuan refugees (n=40) drawn from a wide range of socio-demographic backgrounds (ages, gender, education and roles in the community) both from Kiunga (n=20) and Port Moresby (n=20) communities (1). As expected, no participant was familiar with the formal nomenclature used to identify the index mental disorders under study, although there was a high level of recognition of constituent symptoms and their significance as a source of distress.

Terms for symptoms of PTSD in the lingua franca, Bahasa Indonesian included “waspada” (hypervigilance), “menghindari” (avoidance), “kehilangan minagt” (loss of interest), “dijaga” (startle response), “sakit hati” (anger and resentment), and “tidak percaya” (loss of trust). Similarly, CPTSD symptoms were recognized and named, for example, anger outbursts (“naik dadah”), self-blame (“meduduh diri”), detachment (“tersendiri”), and loss of interest (“kehilangan minat”). Qualitative and psychometric data concerning the reliability and validity of the other categories (MDD, GAD, PD, SAD, CG, and IED) have been reported in previous studies (2-6).

A concordance study conducted with a subsample of respondents from the Port Moresby community found sound agreement on a broad assignment of psychiatric caseness comparing the R-MHAP and the relevant modules of the Structured Clinical Interview for DSM-IV/5 disorders applied in a blinded manner by a psychologist (Area Under the Curve [AUC] 0.93, sensitivity 0.98, specificity 0.97, positive predictive power 0.95, negative predictive power 0.98) (1). Symptoms of the key disorders were relatively stable over six months. Internal reliability was high for the constituent items of all categories: CPTSD (α=.95), PTSD (α=.97), MDD (α=.94), GAD (α=.95), PD (α=.96), SAD (.97), CG (α=.98), IED (α=.93).

The WHODAS has been widely used across epidemiological surveys worldwide, yielding sound internal consistency (Cronbach α=.87 for the entire scale) and test-retest reliability (intraclass correlation coefficients ranging from .69 to .89 across items) in a cross-national survey. The measure has also been applied in clinical studies as a measure of long-term functioning (7). We derived a mean score of functional impairment based on an average of all items endorsed.

**Supplementary file 3a: field personnel training and**

Field interviewers were selected based on their fluency in Bahasa Indonesian, English, and Tok Pisin (the pigeon English widely spoken throughout PNG); their status of trust in the community; and their competence and commitment to the project. The team included an equal number of men and women. The team received three weeks of intensive training under supervision of a clinical psychologist who speaks Bahasa Indonesian, the lingua franca of West Papuans. The training program, applied successfully in the preceding Port Moresby study, focused on interviewing techniques, identification of mental health issues amongst trauma survivors, role-play, and administration of the assessment protocol.

Inter-rater reliability tested in the previous Port Moresby study achieved a 95% overall percentage agreement between field workers and professional personnel in assigning diagnoses. Following training, weekly supervision sessions were conducted via telephone or on-site by the professional team with field workers for the entire study period, extending from March to September 2016.

**Supplementary file 3b: analytic steps and procedure**

We excluded cases with substantial missing data (>25%) on any symptom variables.

T-test and chi-square tests were applied to assess group differences in sociodemographic variables, mean prevalence of CAs, TEs, PLMDs, and functional impairment, comparing the CPTSD, any CMD and no disorder groups. Multivariate analysis of variance (MANOVA) was conducted to examine for a pattern of overall difference (CPTSD vs combined CMD vs no disorder) across groups on relevant variables (CAs, TEs, PMLDs, and functional impairment), while controlling for the independent and interaction effects of the socio-demographic factors (age, gender, marital status, employment, country of birth). The analysis was followed-up with bivariate contrasts (test of mean differences) to examine in more detail the differences between the three groups.

We then derived a summary CPTSD symptom score by adding positively endorsed items to form the dependent variable. We conducted a hierarchal regression analysis, entering blocks of predictor variables in the following stepwise manner: Step 1: sociodemographic variables (age, gender, educational attainment, marital status, employment); Step 2. CA.1 emotional/sexual abuse, CA.2 community violence, CA.3 peer violence; Step 3. mean TE score; and Step 4. mean PMLD mean score. Statistically significant variables at each step (P<.20) were retained in the model. We report 95% confidence intervals.

**Supplementary file 4a:** **Exposure to childhood adversities (CAs) amongst West Papuan refugees from Kiunga (n=486).**

| **Childhood adversities (CAs)** | **N** | **%** |
| --- | --- | --- |
| **Physical/emotional/sexual abuse by parent/guardian** |  |  |
| Verbal abuse or humiliation | 154 | 39.7 |
| Abandoned and thrown out of the house | 63 | 16.3 |
| Physical abuse | 110 | 28.9 |
| Physically assaulted with an object (knife, bottle, stick, whip, club) | 79 | 20.6 |
| Sexual molestation | 32 | 8.7 |
| Forced to perform sexual acts on others | 33 | 9.1 |
| Attempted rape | 28 | 7.7 |
| Rape | 28 | 7.7 |
| At least one |  |  |
| **Community violence** |  |  |
| Witnessing violence in the community | 361 | 78.5 |
| Witnessing someone being stabbed or shot in real life | 230 | 50.4 |
| Witnessing someone being threatened with a knife or gun in real life | 219 | 47.7 |
| **Peer violence** |  |  |
| Bullied by other people | 114 | 32.1 |
| Involved in physical fights with other people | 112 | 29.9 |

**Supplementary file 4b. Exposure to conflict-related traumatic events (TEs) amongst West Papuan refugees from Kiunga (n=486).**

| **Conflict-related TEs** | **N** | **%** |
| --- | --- | --- |
|  |  |  |
| Exposure to combat situations | 121 | 24.9 |
| No shelter | 121 | 24.9 |
| Home destruction | 103 | 21.2 |
| Displaced by conflict | 120 | 24.7 |
| Forced to live in poor conditions | 135 | 27.8 |
| No food or water | 71 | 14.6 |
| Imprisonment | 59 | 12.1 |
| Humiliated | 77 | 15.9 |
| Former combatant | 55 | 11.3 |
| Physical injury | 33 | 6.8 |
| Forced into military | 31 | 6.4 |
| Witnessing murders of family or friends | 68 | 14 |
| Witnessing murders of others | 80 | 16.5 |
| Loss of family members | 96 | 20 |
| Disappearances | 87 | 17.9 |
| Forced to abandon family | 105 | 21.6 |
| Unable to perform proper burials for the deceased | 43 | 8.9 |
| Forced separation | 79 | 16.3 |
| No access to medical care | 119 | 24.5 |
| Torture | 38 | 7.8 |
| Witnessing torture | 189 | 38.9 |
| Witnessing rape or sexual abuse | 41 | 8.4 |
| Witnessing dead bodies | 84 | 17.3 |

**Supplementary file 4c. Exposure to Post-Migration Living Difficulties (PMLDs) amongst West Papuan refugees from Kiunga (n=486).**

| **Post-Migration Living Difficulties** | **N (%)** | **N (%)** | **N (%)** | **N (%)** |
| --- | --- | --- | --- | --- |
|  | **No problem** | **A bit of a problem** | **Moderately serious** | **A very serious problem** |
| Drinking water | 6 (1.2) | 39 (8) | 104 (21.4) | 337 (69.3) |
| Food | 7 (1.4) | 28 (5.8) | 116 (23.9) | 335 (68.9) |
| Shelter | 20 (4.1) | 73 (15) | 120 (24.7) | 273 (56.2) |
| Access to toilet | 90 (18.5) | 148 (30.5) | 118 (24.3) | 130 (26.8) |
| Hygiene | 117 (24.1) | 173 (35.6) | 127 (26.1) | 69 (14.2) |
| Access to clothes, shoes, bedding, blankets | 37 (7.6) | 178 (36.6) | 160 (32.9) | 111 (22.8) |
| Income or livelihood | 7 (1.4) | 31 (6.4) | 65 (13.4) | 383 (78.8) |
| Physical health | 85 (17.5) | 136 (28) | 119 (24.5) | 146 (30) |
| Access to healthcare | 72 (14.8) | 147 (30.3) | 127 (26.1) | 140 (28.8) |
| Distress | 191 (39.4) | 78 (16.1) | 120 (24.7) | 96 (19.8) |
| Safety | 112 (23.1) | 106 (21.8) | 118 (24.3) | 150 (30.9) |
| Access to education | 49 (10.1) | 64 (13.2) | 162 (33.3) | 211 (43.4) |
| Care of family members | 94 (19.3) | 137 (28.2) | 136 (28) | 119 (24.5) |
| Support from others | 73 (15.1) | 95 (19.6) | 103 (21.2) | 214 (44.1) |
| Separation from family members | 172 (35.4) | 113 (23.3) | 82 (16.9) | 119 (24.5) |
| Being displaced from home | 199 (41) | 99 (20.4) | 74 (15.2) | 114 (23.5) |
| Access to information | 104 (21.4) | 113 (23.3) | 132 (27.2) | 137 (28.2) |
| Access to aid | 45 (9.3) | 39 (8) | 41 (8.4) | 361 (74.3) |
| Humiliation or mistreatment | 83 (17.1) | 110 (22.6) | 147 (30.3) | 146 (30) |
| Frequent relocations due to climate and geographic conditions | 101 (20.8) | 145 (29.8) | 135 (27.8) | 105 (21.6) |
| Discrimination | 77 (15.8) | 89 (18.3) | 135 (27.8) | 185 (38.1) |
| Law and order in the community | 85 (17.5) | 152 (31.3) | 110 (22.6) | 139 (28.6) |
| Safety or protection for women from violence in the community | 80 (16.5) | 134 (27.6) | 94 (19.4) | 177 (36.5) |
| Alcohol or drug use in the community | 69 (14.2) | 106 (21.8) | 101 (20.8) | 210 (43.2) |
| Mental illness in the community | 148 (30.5) | 93 (19.1) | 82 (16.9) | 163 (33.5) |
| Care for people in your community who are on their own | 56 (11.5) | 91 (18.7) | 100 (20.6) | 239 (49.2) |

**Supplementary file 5. CPTSD items included in the RMHAP.**

| **RMHAP CPTSD items** | **Content** |  |
| --- | --- | --- |
|  |  |  |
| ***Domain*** | ***Intrusion*** |  |
| **2** | **Distressing dreams** |  |
| **3** | **Flashbacks** |  |
| ***Domain*** | ***Avoidance*** |  |
| **6** | **Internal avoidance** |  |
| **7** | **External avoidance** |  |
| ***Domain*** | ***Hyperarousal*** |  |
| **18** | **Startle response** |  |
| **19** | **Hypervigilance** |  |
| ***Domain*** | ***Affective dysregulation*** |  |
| **16** | **Anger outbursts** |  |
| **11** | **Negative emotions** |  |
| ***Domain*** | ***Negative self-concept*** |  |
| **9** | **Distorted beliefs about self or others** |  |
| **10** | **Self-blame** |  |
| **Domain** | **Interpersonal problems** |  |
| **13** | **Detachment** |  |
| **14** | **Difficulty experiencing positive emotions** |  |

References

1. Tay AK, Rees S, Chen J, Kareth M, Mohsin M, Silove D. The Refugee-Mental Health Assessment Package (R-MHAP); rationale, development and first-stage testing amongst West Papuan refugees. International journal of mental health systems. 2015; 9(1): 1-13.

2. Tay AK, Rees S, Chen J, Kareth M, Lahe S, Kitau R, et al. Associations of Conflict-Related Trauma and Ongoing Stressors with the Mental Health and Functioning of West Papuan Refugees in Port Moresby, Papua New Guinea (PNG). PLoS One. 2015; 10(4): e0125178.

3. Tay AK, Rees S, Chen J, Kareth M, Silove D. The structure of post-traumatic stress disorder and complex post-traumatic stress disorder amongst West Papuan refugees. BMC Psychiatry. 2015; 15(1): 1.

4. Tay AK, Rees S, Chen J, Kareth M, Silove D. The coherence and correlates of intermittent explosive disorder amongst West Papuan refugees displaced to Papua New Guinea. J Affect Disord. 2015; 177: 86-94.

5. Tay AK, Rees S, Chen J, Kareth M, Silove D. Factorial structure of complicated grief: associations with loss-related traumatic events and psychosocial impacts of mass conflict amongst West Papuan refugees. Soc Psychiatry Psychiatr Epidemiol. 2016; 51(3): 395-406.

6. Tay AK, Rees S, Kareth M, Silove D. Associations of Adult Separation Anxiety Disorder With Conflict-Related Trauma, Ongoing Adversity, and the Psychosocial Disruptions of Mass Conflict Among West Papuan Refugees. Am J Orthopsychiatry. 2016.

7. Bottlender R, Strauss A, Moller HJ. Association between psychopathology and problems of psychosocial functioning in the long-term outcome of patients diagnosed with schizophrenic, schizoaffective and affective disorders. Eur Arch Psychiatry Clin Neurosci. 2013; 263(2): 85-92.
